# Automated gait analysis indicates efficacy of T-type calcium channel inhibition for mitigation of disrupted calcium signalling in an SCA5 mouse model

**DOI:** 10.1038/s41598-025-05511-1

**Published:** 2025-07-01

**Authors:** Daumante Suminaite, Nurul Fatihah Talib, Sophie Jane Pettegree, Han Chew Gaelan Tan, Yvonne Louise Odey, Emma Margaret Perkins, Alastair Robert Lyndon, Paul Andrew Skehel, Mandy Jackson

**Affiliations:** 1 The Centre for Discovery Brain Sciences, The University of Edinburgh, Hugh Robson Building, George Square, Edinburgh, EH8 9XD UK; 2 Institute of Life and Earth Sciences, Heriot-Watt University, John Muir Building, Riccarton, Edinburgh, EH14 4AS UK; 3 Euan MacDonald Centre for MND Research, The University of Edinburgh, Edinburgh, EH16 4SB UK

**Keywords:** Neuroscience, Diseases

## Abstract

Spinocerebellar ataxias (SCAs) are dominantly inherited progressive neurodegenerative disorders which to date have no effective treatment. SCA5 arises from mutations in the β-III spectrin gene (*SPTBN2*) with mice lacking β-III spectrin function (β-III^−/−^) mirroring the human clinical phenotype. This study finds evidence for dysregulated calcium homeostasis in β-III^−/−^ mice as evidenced by enhanced auto-phosphorylation of calcium-calmodulin dependent kinase II (CaMKII), a major calcium sensor in cells, and phosphorylation of several CaMKII targets. Mibefradil, an inhibitor of calcium channels, was also found to improve disordered β-III^−/−^ Purkinje cell dendritic morphology in vitro. However, key to evaluation of potential treatments in vivo is the ability to assess relevant changes at later stages of disease in β-III^−/−^ mice, which has previously been problematic. Here the CatWalk XT system was successful in detecting differences in both truncal stability and interlimb coordination across the disease course of β-III^−/−^ mice. Building on these findings CatWalk analysis showed trimethadione, a selective T-type calcium channel inhibitor, but not riluzole nor verapamil, significantly improved interlimb coordination of 8-month-old β-III^−/−^ mice. These findings highlight the CatWalk XT system as a valuable tool to assess age-dependence of motor function and that modulation of T-type calcium channels has therapeutic potential for SCAs.

**Supplementary Information:**

The online version contains supplementary material available at 10.1038/s41598-025-05511-1.

## Introduction

Spinocerebellar ataxias (SCAs) are a genetically heterogenous group of dominantly inherited neurodegenerative disorders, with over fifty genetic loci identified to date^1^. The similarity in clinical presentation of SCAs, of postural abnormalities, progressive motor incoordination and cerebellar degeneration, has led to the belief there are common downstream cellular mechanisms and therefore overlapping treatment options^2^.

SCA type 5 (SCA5) arises from mutations in the gene encoding β-III spectrin (*SPTBN2*)^3–6^, a cytoskeletal protein that is most abundantly expressed in cerebellar Purkinje cell neurons^7^. A mouse line defective in β-III spectrin function (β-III^−/−^ mice) mirrors the human symptoms of SCA5: gait abnormalities, tremor, progressive motor deficits and loss of Purkinje cells^8^. This makes β-III^−/−^ mice a useful preclinical tool to identify new therapeutic avenues for SCAs, a key priority since no current treatments are available to halt or even slow disease progression^9^.

Previous work identified that, downstream of β-III spectrin loss, there are changes to the expression and distribution of specific plasma membrane proteins, in particular the two predominant cerebellar excitatory amino acid transporters (EAAT4 in Purkinje cells and GLAST/EAAT1 in Bergmann glia)^8,10,11^. A direct interaction between β-III spectrin and EAAT4 is required for both EAAT4’s trafficking to and maintenance at the plasma membrane^2,7^. Thus, one immediate consequence of loss of β-III spectrin in Purkinje cells is loss of EAAT4 function^8,12^. Another is that dendrites are thinner, impacting cable properties^13,14^. Together, these early differences lead to β-III^−/−^ Purkinje cell hyperexcitability in young animals, which precedes visible pathological signs of glutamate-mediated excitotoxicity^8^. In the absence of EAAT4, response to synaptic glutamate is greater, eliciting larger amplitudes for parallel fiber-mediated excitatory postsynaptic currents (PF-EPSCs)^8,12^ and in β-III^−/−^ Purkinje cells, less depolarising current is required, compared to wildtype, for repetitive firing of action potentials, indicating membranes are closer to threshold^15^. In contrast, from 3-months of age progressive loss of GLAST is observed in β-III^−/−^ mice, correlating with an age-dependent accumulation of degenerating Purkinje cells, the majority exhibiting ultrastructural signs of glutamate-mediated excitotoxicity^2,8,12^. These findings suggest Purkinje cell hyperexcitability in β-III^−/−^ mice is upstream of a non-cell autonomous mechanism of glutamate-mediated excitotoxicity, with the latter culminating in cell death. The pertinent molecular changes orchestrating such upstream hyperexcitability processes are yet to be determined. Yet this understanding will facilitate the development of effective treatments as targeting direct upstream pathological triggers, rather than adaptive responses to counteract neuronal dysfunction, will increase the likelihood of positively impacting the disease process.

A common feature in several SCAs is disrupted calcium homeostasis^16^. Such a mechanism could be at play early in the disease course of SCA5 since EAAT4 is known to constrain the activation of mGluR1^15,17^, an upstream regulator of type 1 inositol 1,4,5-trisphosphate receptor (IP_3_R1)-mediated calcium signalling^18^ and low-voltage gated channels such as T-type calcium channels may be more readily activated in the absence of β-III spectrin function due to dendrites being closer to threshold^13,15^.

Here we report the first molecular evidence for early dysregulated calcium homeostasis in β-III^−/−^ mice. It also establishes the use of automated video-assisted gait analysis (Noldus CatWalk XT system) as a means to effectively monitor the age-dependence of motor function in SCA rodent models and to detect locomotor improvement following treatment at later symptomatic stages, identifying modulation of T-type calcium channels as a promising novel therapeutic strategy across the disease course.

## Materials and methods

### Animals

Animal work was performed in accordance with the UK Animal (Scientific Procedures) Act 1986 and approved by the University of Edinburgh Animal Welfare and Ethical Review Body. The authors complied, where feasible, with the ARRIVE guidelines. Mice of both sexes were housed with same-sex littermates in a regulated environment (temperature 22 ± 2 °C; humidity 40 ± 10%) under a 14 h light/10 h dark cycle with *ad libitum* access to food and water. The β-III spectrin-deficient mouse line (β-III^−/−^) generated on a C57BL6 background at The University of Edinburgh^8^ was bred in-house. All bred mice (wild type and β-III^−/−^) possess an eGFP reporter transgene having been crossed with EAAT4-BAC-eGFP transgenic mice (C57BL6 background) obtained from Johns Hopkins University^75^.

### Immunoblotting

Cerebella collected post-euthanasia by isoflurane inhalation were homogenised in 20 mM HEPES, pH 7.4, 1 mM sodium orthovanadate, 1 µg/ml protease inhibitor cocktail using a glass-teflon homogeniser. Protein concentrations were calculated using Coomassie Plus reagent (Pierce) and a range of BSA standards. Lysates were heated for 10 min at 95 °C prior to separation by denaturing SDS-PAGE. Proteins transferred to Hybond ECL nitrocellulose membrane (0.2 μm pore size; Amersham, GE Healthcare) in transfer buffer (25 mM Tris, 187 mM glycine, 20% v/v methanol). Membranes blocked in Tris-buffered saline/Tween20 (TBST) (8 mM Tris, 42 mM Tris-HCl, 200 mM NaCl, 0.1%(v/v) Tween 20) containing 5% (w/v) non-fat dry milk before overnight incubation at 4 °C in primary antibody diluted in blocking buffer or 1% (w/v) BSA for phospho-specific antibodies [1:1000 for rabbit anti-Akt, -pAkt(Ser463), −4E-BP1, p4E-BP1(Thr37/46)(Cell Signalling Technology); mouse anti-CaMKII (1:10,000; Life Technologies, #137300), rabbit anti-pCaMKII(T287)(1:1000; Cell Signalling), -pGluR1(S831) (1:1000 AbCam ab109464). After antibody specificity confirmed on full-length blots, membranes were cut prior to blocking, facilitating the detection of multiple proteins with the same membrane. Membranes washed in TBST before incubation at room-temperature with appropriate horseradish peroxidase-(HRP)-conjugated secondary antibody – goat anti-rabbit or donkey anti-mouse (1:4000, GE Healthcare) diluted in blocking buffer followed by washes in TBST. Protein bands of the correct molecular weight, visualised by enhanced chemiluminescence (BioRad), were quantified using ImageJ software. Anti-VapA (kind gift of Paul Skehel) was used as loading control.

### Dissociated primary cerebellar cultures

Postnatal cultures using P0 mouse pups, anaesthetised with isoflurane before decapitation, were performed adopting previous methods^76,77^. Images were captured with a Zeiss inverted LSM510 laser scanning microscope at The University of Edinburgh IMPACT imaging facility. Number of cell dendritic branches and total dendritic length were obtained through Scholl analysis using NeuronStudio software.

### Automated gait analysis

Gait analysis was performed with the CatWalk automated gait analysis system (Noldus Technology; software version XT 8.1) and the following parameters analyzed: body speed, base of support, phase dispersion, print position, regularity index and support. Glass plate was elevated 1.5 m above the floor with side panels adjusted to 3.5 cm to deter turning whilst preserving free movement. Mice were habituated to the apparatus by placing the animals in the walkway and allowing free exploration for approximately 10 min on two consecutive days. Habituation and recordings were performed by the same researcher. Pawprints were captured by a high-speed camera (GP-2360c, GEViCam) positioned below the glass plate and runs recorded at 100 frames-per-second. All recordings were performed in a darkened room with camera gain and intensity threshold optimised for each age group to minimise background and maximise paw detection. Each animal completed 3–5 compliant runs in a single trial. Compliant run criteria were defined as a run between 0.5 and 5.0 s in duration for young animals and between 0.5 and 8.0 s for old animals. With age blinding to genotype was not possible to maintain due to observed differences in the walking behaviour of control and β-III^−/−^ mice. The CatWalk XT 8.1 software was used to automatically classify all pawprints but each pawprint was visually inspected to confirm the correct classification and any error manually corrected, with more mis-classifications generated for β-III^−/−^ mice. The walkway was cleaned as required to remove debris, using 70% ethanol and dried before resuming recording.

### Drug preparation and dosing

All drugs were purchased in powdered form and reconstituted according to manufacturer’s instructions: trimethadione (Merck Sigma-Aldrich; T0781), riluzole, 98% (Acros Organics; 459360010), verapamil hydrochloride (Fluorochem; 047206) and mibefradil (Tocris; 2198). Prior to drug administration, mice were randomly assigned, weighed, and water consumption measured over 5 days to determine requisite amount of drug stock solutions to be added to 100 ml of water to achieve the required final consumption when drinking normally. Following drug administration water consumption was monitored for 4 days for each cage to ensure maintenance of the correct dosage of drug. Doses chosen were based on previous studies^37,53,54,61–63^. Water for both drug and untreated β-III^−/−^ mice contained 0.2% (w/v) sucrose to encourage drinking.

### Statistical analysis

Normality of data was tested using Q-Q plots and Shapiro-Wilk test. A sub set of CatWalk data was not normally distributed, so for consistency all statistical analyses were performed using non-parametric tests. Two groups defined by a single independent variable were analysed with Mann-Whitney U test and three groups defined by a single independent variable were analysed with Kruskal-Wallis H test followed by Bonferroni multiple comparison correction. All statistical analysis performed using SPSS statistics software version 29 (IBM). All graphs presented as median, 25th and 75th percentiles, minimum and maximum, with mean represented with cross. Sample size (N) denotes the number of independent animals or individual cultures and (n) denotes number of individual cells. Indication of significance is as follows: *p < 0.05, **p < 0.01, ***p < 0.001 between groups; ^#^*p* < 0.05, ^##^*p* < 0.01, ^###^*p* < 0.001 between β-III^−/−^ age groups; ^≠^*p* < 0.05, ^≠≠^*p* < 0.01, ^≠≠≠^*p* < 0.001 between control age groups.

## Results

### Early aberrant calcium homeostasis in β-III^−/−^ mice

**Fig. 1:**
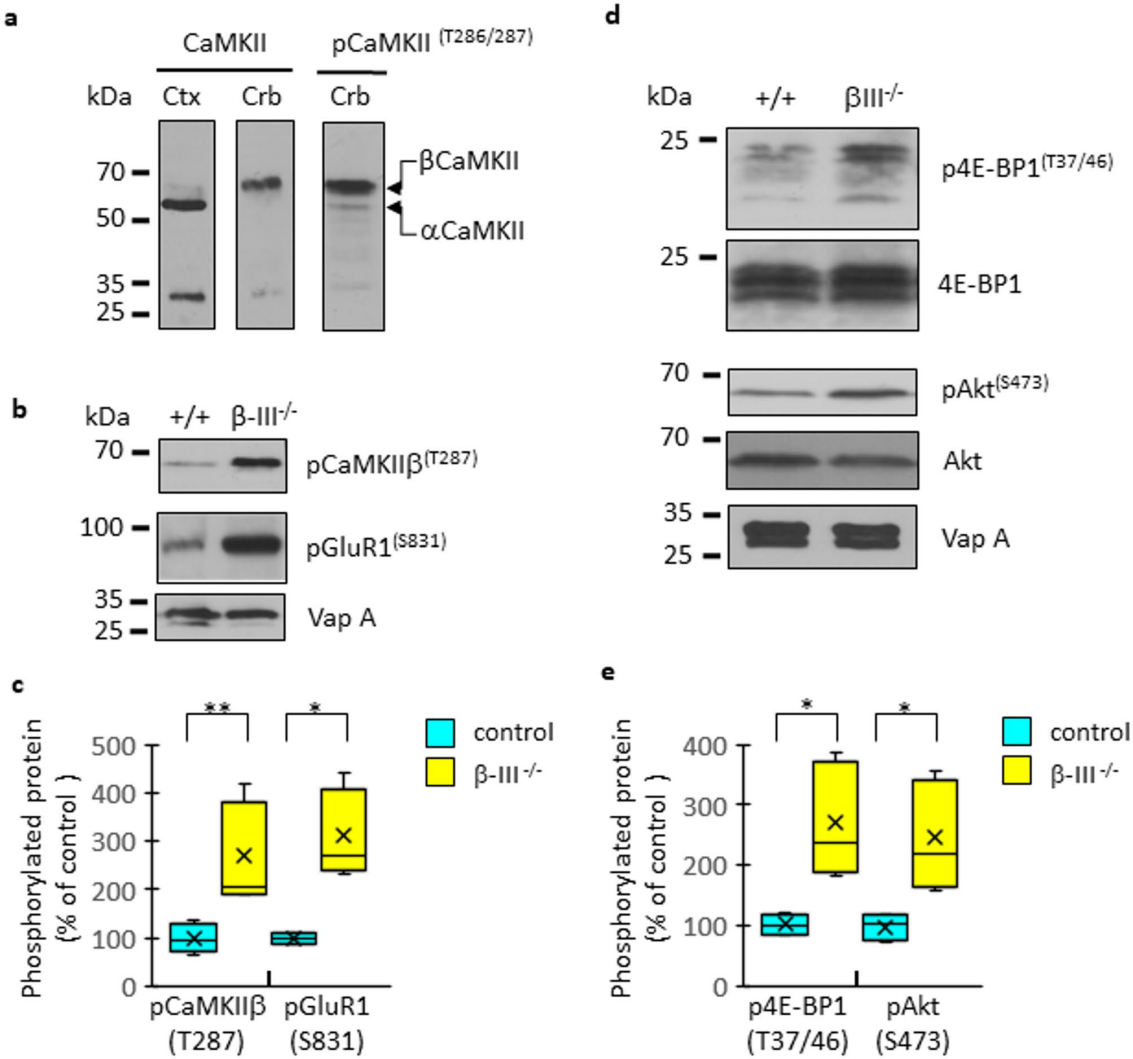
Disrupted CaMKII signalling in young β-III^−/−^ mice. (**a**) Immunoblots of cortical and cerebellar lysates from 6-week-old mice, probed with either a pan antibody against CaMKII or phospho-specific CaMKII antibody. Higher molecular weight β isoform more abundant in cerebellum whilst α isoform more abundant in cortex. See supplementary Fig. 1a for full-length blots. (**b**) Cropped immunoblots of control (+/+) and β-III^−/−^ cerebellar lysates from 6-week-old mice probed with phospho-specific antibodies against CaMKII and a CaMKII target, GluR1. See supplementary Fig. 1b for full-length blots. VapA used as internal loading control. (**c**) Quantification of elevated protein phosphorylation in β-III^−/−^ (yellow) cerebellum as a percentage of control (blue) (*N* = 5-6). (**d**) Cropped blots examining phosphorylation status of two effectors of a CaMKII activated signalling cascade, Akt (protein kinase B) and 4E-BP1. See supplementary Fig. 1c for full-length blots. VapA used as internal loading control. (**e**) Quantification of enhanced protein phosphorylation in cerebellar lysates from 8-week-old β-III^−/−^ mice (yellow) as a percentage of control (blue) (*N* = 4).

Given the possibility that disrupted calcium homeostasis, mediated by inefficient glutamate clearance and abnormal dendritic morphology, could be an early factor in SCA5 pathogenesis, this study first investigated whether aberrant calcium-mediated kinase activity is a cellular consequence of β-III spectrin loss. One such enzyme is calcium-calmodulin dependent kinase II (CaMKII), a major calcium sensor in cells, composed of 12–14 subunits^19–21^. In brain the CaMKII holozyme consists of α and β subunits with rises in intracellular calcium resulting in its activation and autophosphorylation at T286 (on α subunits) or T287 (on β subunits)^22^. CaMKIIβ is reported to be the major isoform in the cerebellum^23^ and through its binding to actin filaments plays a critical role in modulating the subcellular localization of the CaMKII holozyme^24^. Immunoblotting with an antibody that recognizes both α and β subunits corroborated CaMKIIβ as the main isoform in our cerebellar lysates (Fig. 1a). Using a phospho-specific antibody, a two-fold increase in phosphorylation of CaMKIIβ was observed in the cerebellar lysates of young β-III^−/−^ mice compared to age-matched controls (Fig. 1b, c & Supplementary Fig. 1; *N* = 6, *p* = 0.002). Similarly, increased phosphorylation of a downstream CaMKII target, the GluR1 subunit of AMPA receptors^25,26^, was detected in young β-III^−/−^ mice (Fig. 1b & c; *N* = 5, *p* = 0.036). Two key proteins (protein kinase B (Akt) and 4E-BP1) within the mTOR signalling pathway, a CaMKII activated cascade^27^, were also found to be excessively phosphorylated in young β-III^−/−^ mice (Fig. 1d & e). Levels of p4EBP1 and pAkt were both two-fold greater than controls, *p* = 0.029, *N* = 4). Together these findings in young β-III^−/−^ mice support excessive calcium signalling as an early feature of SCA5 pathogenesis.

**Fig. 2:**
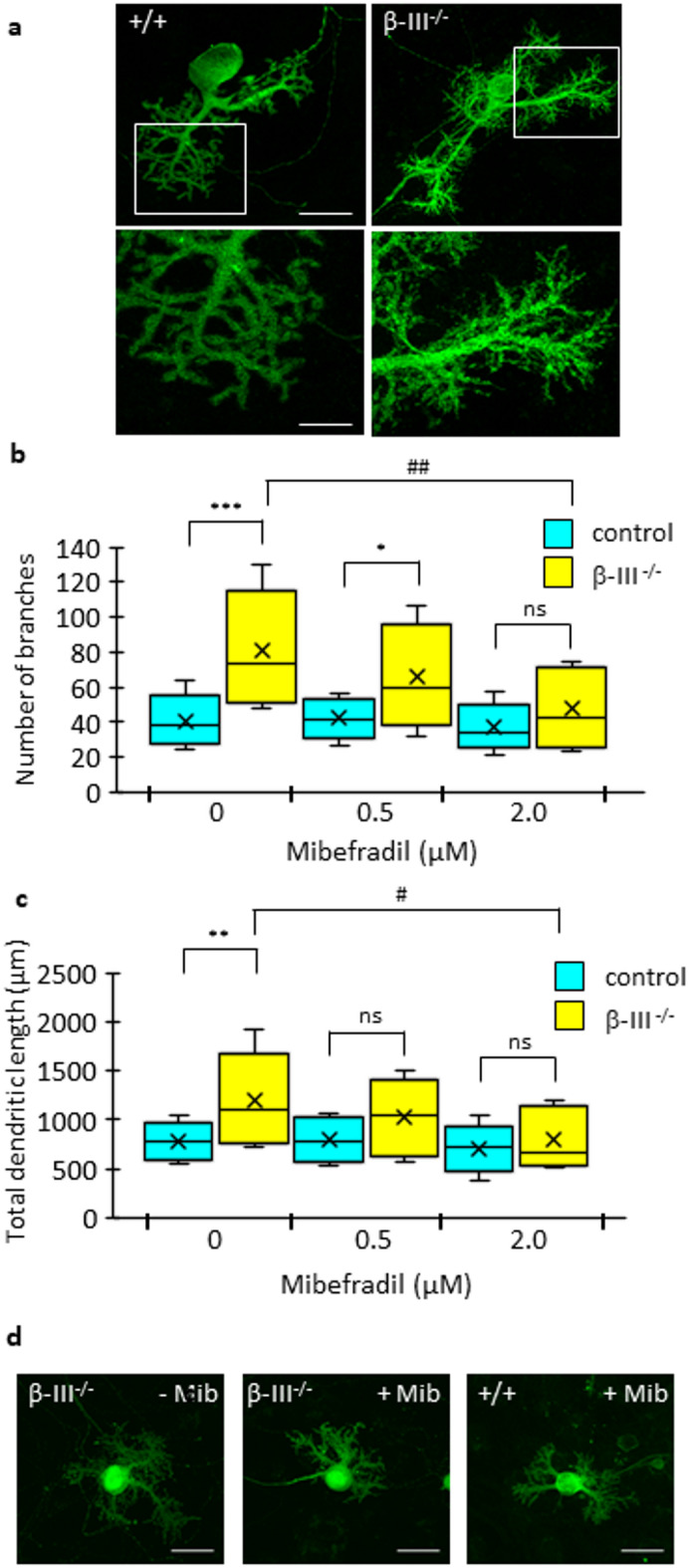
In vitro mibefradil treatment ameliorates aberrant dendritic morphology of β-III^−/−^ Purkinje cells. (**a**) Dissociated control (+/+) and β-III^−/−^ cerebellar cultures, maintained for 21 days in vitro, immunostained for IP_3_R1, a Purkinje cell specific marker. Representative image shows excessive “feathery” dendritic morphology of β-III^−/−^ Purkinje cells. Scale bar, 50 μm. Lower panels show higher magnification of boxed area. Scale bar, 25 μm. (**b**) Quantification of number of Purkinje cell dendritic branches in 12-day-old dissociated control (+/+; blue) and β-III^−/−^ (yellow) cerebellar cultures, in the absence or presence of mibefradil (*N* = 3 for both genotypes; *n* = 8–17 for each condition). Number of branches per Purkinje cell significantly reduced for β-III^−/−^ cultures (yellow) treated with 2 µM mibefradil compared to 0 µM mibefradil, denoted by ##. Statistical differences between control (blue) and β-III^−/−^ (yellow) cultures denoted by asterixis (*). (**c**) Quantification of total Purkinje cell dendritic length in 12-day-old dissociated control (+/+; blue) and β-III^−/−^ (yellow) cerebellar cultures, in the absence or presence of mibefradil (*N* = 3 for both genotypes; *n* = 8–17 for each condition). Total dendritic length per Purkinje cell significantly reduced for β-III^−/−^ (yellow) cultures treated with 2 µM mibefradil compared to 0 µM mibefradil, denoted by #. Statistical differences between control (blue) and β-III^−/−^ (yellow) cultures denoted by asterixis (*). All data plotted as median, quartiles (25th and 75th percentiles), minimum and maximum with mean indicated by cross. (**d**) Representative images of β-III^−/−^ Purkinje cells cultured for 12-days in the absence or presence of 2 µM mibefradil, with latter showing dendritic morphology similar to mibefradil treated control (+/+) Purkinje cell. Scale bar, 25 μm.

To further assess whether aberrant calcium signalling is an early outcome of β-III spectrin loss in vitro experiments were carried out on dissociated cerebellar cultures. Previous in vivo work had shown that prior to undergoing degeneration β-III^−/−^ Purkinje cells exhibit aberrant morphology including thinner dendrites, loss of planarity and abnormal branching^13^. By immunostaining cultures for IP_3_R1, a Purkinje cell marker, a dis-ordered “feathery” appearance of Purkinje cells lacking β-III spectrin was evident in 21-day-old dissociated cerebellar cultures, illustrating the observed in vivo aberrant morphology is recapitulated in vitro (Fig. 2a). Given the pivotal role calcium signalling plays in dendritic development^28^ and the fact overexpression of CaMKIIβ has been shown to enhance neurite extension^29^ we addressed whether this dis-ordered dendritic morphology could be associated with excessive calcium signalling. Dissociated cerebellar cultures were treated for 12-days with mibefradil, a widely reported, broad spectrum calcium channel blocker, inhibiting both L-type and low-voltage-activated T calcium channels^30^. Number of branches (Fig. 2b) and total dendritic length (Fig. 2c) were quantified revealing cultured β-III^−/−^ Purkinje cells had 192% more dendritic branches and 144% greater dendritic length than control cells (+/+, 38 (30.5, 47); β-III^−/−^ 73 (54, 100) branches/cell, *p* = 2.58E-06; +/+, 771.3 (633.1, 906.7); β-III^−/−^ 1110.4 (807.9, 1412) µm of dendrite, *p* = 0.002). The increase in the number of dendritic branches of β-III^−/−^ Purkinje cells was significantly reduced when cultured for 12 days in the presence of 2 µM mibefradil (Fig. 2b; *p* = 0.009). Moreover, the difference in dendritic branches between β-III^−/−^ and control Purkinje cells was abolished by 2 µM mibefradil (Fig. 2b & d; control 35 (31, 43); β-III^−/−^ 43 (29, 68) branches/cell *p* = 0.195). Similarly, total dendritic length of β-III^−/−^ Purkinje cells was significantly reduced in the presence of 2 µM mibefradil (Fig. 2c; *p* = 0.016), resulting in no significant difference to control cells exposed to 2 µM mibefradil (Fig. 2c & d; control, 717.3 (595.5, 801.3) µm; β-III^−/−^, 665.7 (576.4, 1074.9) µm; *p* = 0.544). Neither 0.5 nor 2 µM mibefradil had any significant impact on control Purkinje cell morphology. Together these findings support elevated calcium signalling as playing a role in neuronal dysfunction in β-III^−/−^ mice and therefore as a potential therapeutic target.

### CatWalk XT system in monitoring progressive spinocerebellar ataxia

Key to being able to evaluate potential treatments based on the above findings is the ability to monitor relevant locomotor parameters across the disease course. Once overt symptoms have emerged accurate quantification of locomotor characteristics is hampered by compromised engagement with behavioural tests. Motor behaviour of β-III^−/−^ mice has been measured using inked footprint, rotarod and elevated beam analyses^8^. However, the gait and locomotor parameters that were assessed using these methods were limited. Moreover, by 6-months of age, β-III^−/−^ mice were unable to carry out the rotarod, even at the lowest speed and were very hesitant in crossing an elevated beam^8^. This makes it difficult to assess relevant changes in motor performance at later stages of disease. In contrast, automated video-assisted gait analysis (Noldus CatWalk XT system), which is less stressful to the animal and measures natural walking behaviour, has the potential to provide quantitative measurements for a variety of spatiotemporal and kinetic parameters across the disease course.

**Fig. 3:**
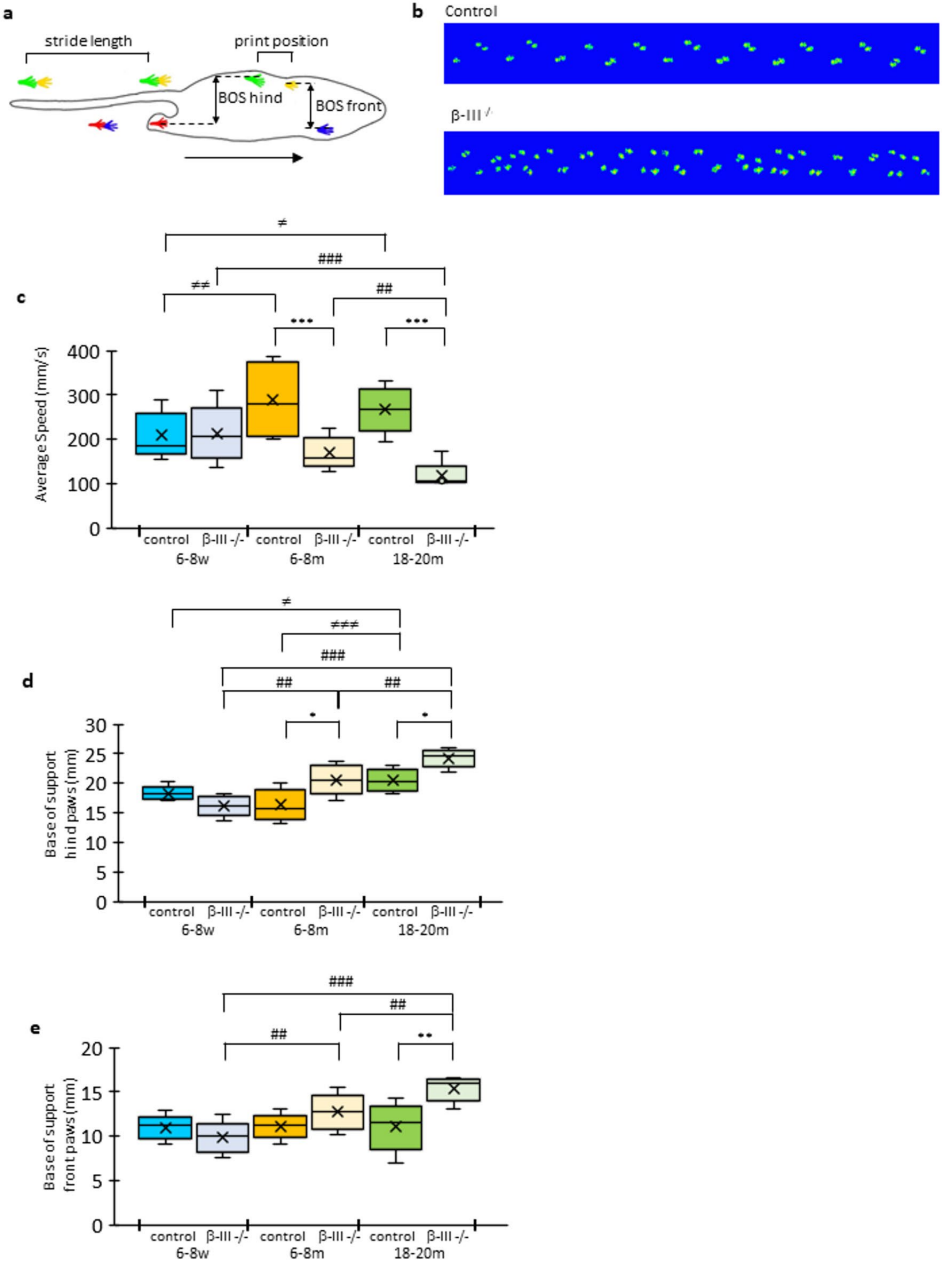
Progressive truncal instability and reduced speed in β-III^−/−^ mice compared to age-matched controls. (**a**) Schematic of different spatial gait parameters obtained with CatWalk XT system: stride length, print position and base of support (BOS). (**b**) Representative CatWalk XT run for control and β-III^−/−^ mice aged 18-months. Quantification of (**c**) average body speed, (d) hind limb base of support and (e) forelimb base of support of control (dark coloured boxes) and β-III^−/−^ mice (light coloured boxes) at 6–8-weeks (6-8w; blue), 6–8-months (6–8 m; orange)- and 18–20-months of age (18–20 m; green). Progressive decrease in body speed and increase in base of support observed for β-III^−/−^ mice, compared to age-matched controls (*N* = 10–16). All data plotted as median, quartiles (25th and 75th percentiles), minimum and maximum with mean indicated by cross. Statistical differences between control and β-III^−/−^ animals of the same age denoted by *; between β-III^−/−^ animals of different ages denoted by #; between control animals of different ages denoted by ≠.

**Fig. 4:**
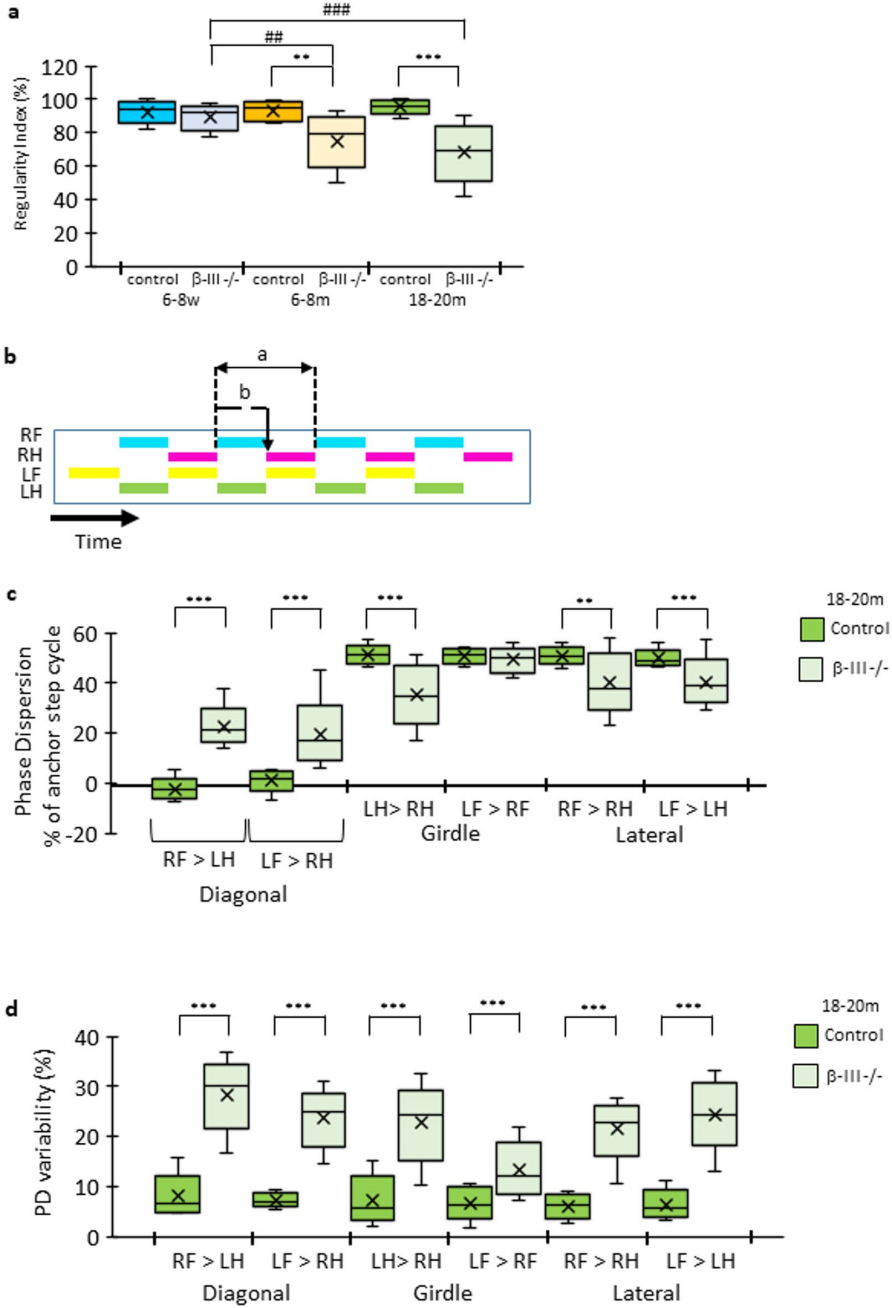
Decrease in interlimb coordination of β-III^−/−^ mice compared to age-matched controls. (**a**) Box plots for regularity index of β-III^−/−^ (light coloured boxes) and control mice (dark coloured boxes) at 6–8-weeks (6-8w; blue), 6–8- months (6–8 m; orange) and 18–20-months (18–20 m; green) of age. Statistical differences between control and β-III^−/−^ animals of the same age denoted by *; between β-III^−/−^ animals of different ages denoted by #. (**b**) Schematic representation of phase dispersion for RF-RH (anchor-target) paws. Calculation is based on “a” the step cycle duration of the anchor paw (RF) and “b” the length of time into the anchor paw’s step cycle when the target paw (RH) makes first contact (initial contact of target paw – initial contact of anchor paw/step cycle of anchor paw x 100%). (**c**) Quantification of average phase dispersion of the six different paw combinations for 18–20-month-old animals. β-III^−/−^ mice (light green), compared to age-matched controls (dark green), have significantly reduced phase dispersion for lateral and girdle paw pairs, except for LF > RF, and significantly increased phase dispersions for diagonal paw pairs (**d**) Quantification of the variability in average phase dispersion of the six paw combinations for 18–20-month-old animals. β-III^−/−^ mice (light green), compared to age-matched controls (dark green), have significantly greater variability for all paw combinations. Data (*N* = 10–16) plotted as median, quartiles (25th and 75th percentiles), minimum and maximum with mean indicated by cross.

**Fig. 5:**
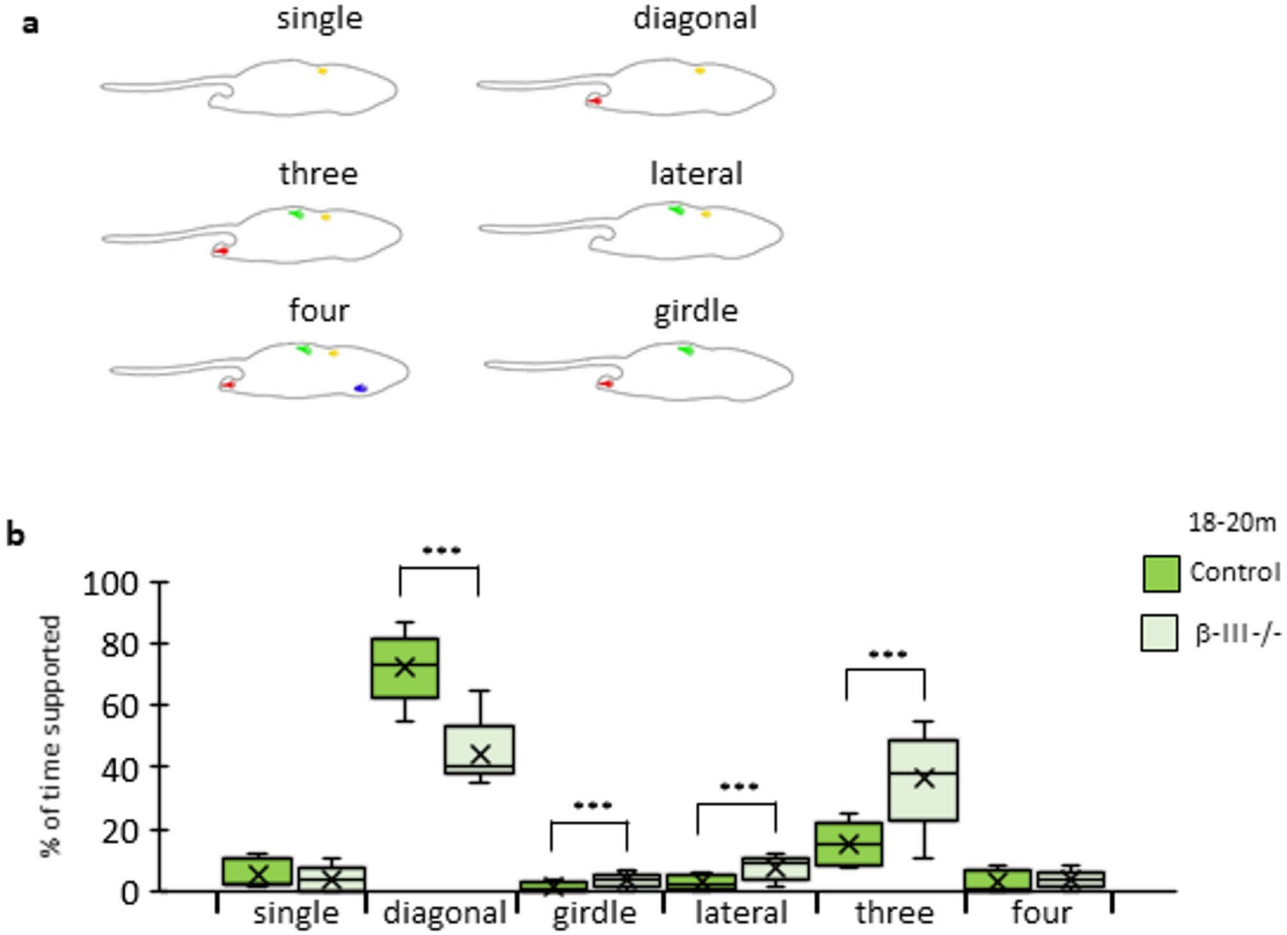
Altered support in β-III^−/−^ mice compared to age-matched controls. (**a**) Schematic representation of the various paw combinations: single, three, four, diagonal (opposite forepaw and hindpaw), lateral (hind and forepaw on the same side) and girdle (both hind or both forepaws). (**b**) Box plots showing the average percentage of total run duration spent supported on the different paw combinations for 18–20-month-old animals. β-III^−/−^ mice (light green) spent significantly less time on diagonal paws than controls (dark green) and more time on their lateral, girdle and three paws. Data plotted as median, quartiles (25th and 75th percentiles), minimum and maximum with mean indicated by cross.

The CatWalk XT system was used to monitor the progression of the motor phenotype of β-III^−/−^ mice, animals aged 6–8-weeks-old, 6–8-months-old, and 18–20-months-old, alongside age-matched control animals. Parameters predicted to be impacted by ataxia and commonly reported in studies characterizing gait disturbances in other preclinical models (reviewed in^31^) were examined (Figs. 3, 4 and 5): body speed, number of steps, stride length (the distance between the placement of a paw and the subsequent placement of the same paw), base of support (BOS; distance between forelimbs or hindlimbs measured perpendicular to direction of walking), print position (distance between the centre of the hind paw and the centre of the previously placed front paw on the same side of the body (ipsilateral) and in the same step cycle), regularity index (RI), phase dispersion (PD) and support time.

### Reduced speed and truncal stability in older β-III^−/−^ mice

In β-III^−/−^ mice, but not control animals, a progressive decrease in average body speed and stride length, with an increased number of steps was observed (Fig. 3c; Table 1). Consistent with previous inked footprint analysis^8^, there was an increase in the hindlimb base of support (BOS) with age, for both β-III^−/−^ and control mice, and at 6–8 and 18–20 months of age there was a significantly wider hindlimb BOS in β-III^−/−^ mice compared to controls (Fig. 3d). Together the reduced stride length and increased hindlimb BOS in 6–8-month and 18–20-month-old β-III^−/−^ mice indicate reduced trunk stability. In addition, CatWalk XT analysis revealed a significantly larger forelimb BOS in β-III^−/−^ mice, compared to controls at 18–20-months of age (Fig. 3e). Print position, another spatial gait parameter, was also found to be significantly different between old β-III^−/−^ and control mice, with the former placing their hindlimbs further away from where the previous ipsilateral forepaw had been placed. This is another sign of motor pathology since to maximise the safe placement of hind paws, rodents normally place their hind paws as close to the previous forepaw as possible. If hind paw is placed behind the forepaw, value is positive whereas value is negative if hind paw placed in front of forepaw: right print position, control 10.15 (7.13, 12.13) mm, β-III^−/−^ 18.21 (16.44, 19.91) mm, *p* = 3.67E-05; left print position, control 9.40 (7.69, 13.25), β-III^−/−^ 21.04 (19.41, 21.51) mm, *p* = 1.38E-04.

**Table 1:** Median, quartiles (25th and 75th percentiles) and statistical results for stride lengths and number of steps at the different ages for control and βIII^−/−^ mice. *p* levels < 0.05 are shown in bold.

Parameter	Group	6–8 wk old mice	6–8 mo old mice	18–20 mo old mice
RF stride length (mm)	control βIII-/- *p*	63.47 (61.53, 66.05) 54.30 (51.05, 63.99) *0.019	65.02 (57.74, 69.44) 53.21 (49.85, 65.37) 0.150	68.43 (60.53, 70.45) 45.30 (41.30, 47.98) ***2.64E-07
RH stride length (mm)	control βIII-/- *p*	59.80 (53.55, 65.39) 57.87 (49.12, 61.51) 0.259	64.15 (59.65, 70.05) 51.49 (47.08, 54.37) ***0.000138	67.85 (61.96, 70.35) 37.05 (34.27, 43.34) ***3.77E-07
LF stride length (mm)	control βIII-/- *p*	62.05 (58.44, 66.09) 57.40 (53.22, 66.56) 0.312	62.04 (57.89, 71.22) 52.36 (47.70, 61.29) *0.012	69.71 (62.45, 70.73) 47.84 (46.71, 49.21) ***4.52E-06
LH stride length (mm)	control βIII-/- *p*	58.34 (55.67, 59.94 59.79 (54.54, 63.75) 0.371	63.28 (56.87, 71.52) 52.14 (43.98, 55.91) ***0.000248	66.64 (61.78, 70.45) 36.23 (35.81, 38.07) ***3.77E-07
Number of steps	control βIII-/- *p*	47.35 (43.3, 49.4) 45 (42.9, 50.7) 0.585	45.15 (40.3, 48.93) 56 (49.38, 58.63) **0.002	41.33 (40.38, 45.75) 71.99 (64.33, 74) ***3.77E-07

RF: right forelimb; LH: left hind limb; LF: left forelimb; RH: right hind limb.

### Decrease in interlimb coordination in β-III^−/−^ mice

Regularity index (RI) is a measure of interlimb coordination and a value of 100% is regarded as a fully coordinated run, where each paw is placed once every fourth step. A lower RI was observed in β-III^−/−^ mice (6–8- and 18–20-month-old animals) compared to age-matched controls, indicating β-III^−/−^ mice have a larger number of missteps interspersing the normal step patterns (Fig. 4a).

Similarly phase dispersion, relating to the time between footfalls is another measurement of interlimb coordination^32^, and is significantly different between β-III^−/−^ and control mice (Table 2; Fig. 4b–d). For phase dispersion, the time of initial contact for one paw (the target) is expressed as a percentage of the stride cycle of a second paw (the anchor) (Fig. 4b). Phase dispersion can therefore be calculated for girdle forepaws or girdle hind paws (LF > RF and LH > RH: with left paws always set as the anchor), for lateral paws, those on the same side (LF > LH and RF > RH) and for diagonal paws (LF > RH and RF > LH). Forepaws are always set as the anchor for phase dispersion of lateral and diagonal paws.

**Table 2:** Median, quartiles (25th and 75th percentiles) and statistical results for phase dispersion of control and βIII^−/−^ mice at three different ages. *p* levels < 0.05 are shown in bold.

Paws	Group	6–8 wk old mice	6–8 mo old mice	18–20 mo old mice
RF > LH	control βIII-/- *p*	4.60 (1.70, 7.08) 5.76 (1.92, 7.67) 0.586	5.61 (1.87, 7.31) 13.73 (6.84, 17.81) **0.003	−2.32 (−5.19, −1.36) 21.31 (18.72, 21.98) ***3.77E-07
LF > RH	control βIII-/- *p*	3.74 (1.64, 5.52) 8.70 (6.83, 11.84) *** 0.001	3.96 (2.89, 6.18) 12.67 (6.65, 17.02) ***0.00025	1.92 (−0.09, 4.58) 17.14 (11.89, 17.8) ***3.77E-07
LH > RH	control βIII-/- *p*	49.77 (48.48, 53.03) 47.72 (45.31, 51.94) 0.097	47.85 (44.78, 50.55) 42.20 (36.87, 47.08) *0.041	51.33 (48.72, 53.04) 34.70 (30.24, 42.81) ***1.13E-05
LF > RF	control βIII-/- *p*	50.18 (49.50, 51.86) 48.89 (46.37, 50.97) 0.310	48.10 (45.82, 51.31) 46.56 (36.56, 48.70) 0.363	51.45 (49.44, 52.91) 50.13 (46.1, 51.34) 0.077
RF > RH	control βIII-/- *p*	52.14 (50.71, 53.27) 50.56 (47.64, 53.88) 0.262	51.80 (46.82, 55.25) 49.73 (42.24, 51.03) 0.182	50.55 (48.81, 52.09) 37.86 (35.11, 46.39) **0.002
LF > LH	control βIII-/- *p*	52.51 (50.16, 53.48) 51.64 (48.57, 54.15) 0.660	52.70 (48.48, 56.60) 46.57 (41.89, 52.74) *0.031	48.79 (47.74, 50.19) 38.94 (35.32, 41.79) ***5.23E-05

RF: right forelimb; LH: left hind limb; LF: left forelimb; RH: right hind limb.

In a coordinated animal moving at moderate speed, phase dispersion should be close to 0% for diagonal paws, as both paws should make contact at roughly the same time. In contrast, the phase dispersion of girdle and lateral paws should be around 50%, with the target paw making contact approximately half-way through the stride cycle of the anchor paw. Analysis shows at 6–8 months of age there is a significant difference in the placement of four paw combinations (RF > LH, LF > RH, LH > RH, LF > LH) for β-III^−/−^ mice compared to control mice (Table 2). At 18–20-months of age, it is only the phase dispersion of the girdle forepaws (LF > RF) that is not significantly different to control mice (Table 2; Fig. 4c). In addition, the variability of phase dispersion within a run is used as another measure of accuracy in inter-limb coordination with little phase dispersion variability expected during coordinated locomotor activity. In 6–8-month-old and 18–20-month-old β-III^−/−^ mice there is significantly more variability in phase dispersion for all six paw combinations compared to control animals, further highlighting aberrant interlimb coordination with age (Table 3; Fig. 4d).

**Table 3:** Median, quartiles (25th and 75th percentiles) and statistical results for the variability in phase dispersion of control and βIII^−/−^ mice at three different ages. *p* levels < 0.05 are shown in bold.

Paws	Group	6–8 wk old mice	6–8 mo old mice	18–20 mo old mice
RF > LH	control βIII-/- *p*	14.03 (8.02, 15.64) 12.76 (9.14, 18.53) 0.421	7.56 (5.82, 10.28) 22.35 (17.43, 25.09) ***7.15E-06	6.61 (4.90, 8.46) 30.00 (26.78, 31.95) ***3.77E-07
LF > RH	control βIII-/- *p*	8.06 (5.50, 13.83) 14.32 (11.65, 22.23) * 0.014	7.06 (5.90, 14.58) 21.21 (15.91, 25.11) ***0.0001	6.86 (6.41, 8.24) 25.05 (21.21, 26.37) ***3.77E-07
LH > RH	control βIII-/- *p*	9.08 (6.54, 14.49) 12.74 (10.29, 19.44) 0.201	6.81 (5.24, 12.39) 19.58 (15.52, 29.84) ***0.0001	5.72 (4.39, 8.96) 24.40 (20.10, 26.10) ***4.52E-06
LF > RF	control βIII-/- *p*	11.06 (7.70, 14.15) 11.10 (9.12, 16.88) 0.737	8.54 (6.52, 16.83) 16.63 (10.64, 25.34) *0.017	6.37 (5.34, 9.10) 12.19 (10.09, 15.75) ***5.23E-05
RF > RH	control βIII-/- *p*	9.91 (6.72, 12.66) 12.94 (9.78, 16.70) 0.077	6.11 (5.14, 9.55) 16.85 (14.12, 20.63) ***0.00043	6.35 (4.47, 7.62) 22.96 (21.55, 24.42) ***3.77E-07
LF > LH	control βIII-/- *p*	9.98 (7.41, 13.39) 11.71 (7.48, 16.10) 0.310	7.51 (5.18, 10.83) 17.22 (14.74, 22.48) ***0.000138	5.69 (4.66, 7.40) 24.44 (23.21, 28.55) ***3.77E-07

RF: right forelimb; LH: left hind limb; LF: left forelimb; RH: right hind limb.

Finally, support time is the percentage of time the animal spends supporting their weight on a particular combination of paws. The combinations are a single paw, two girdle paws, two lateral paws, two diagonal paws, three paws or all four paws (Fig. 5a). The fact rodents typically have a symmetrical gait pattern means that diagonal support normally has the highest value. A significant increase in either girdle, lateral and/or three paw support was observed for β-III^−/−^ mice at all ages compared to controls, with a decrease in diagonal support observed by 6–8-months of age (Table 4; Fig. 5b).

**Table 4:** Percentage of run supported by different paw combinations. Median, quartiles (25th and 75th percentiles) and statistical results presented for control and βIII^−/−^ mice at three different ages. *p* levels < 0.05 are shown in bold.

Support	Group	6–8 wk old mice	6–8 mo old mice	18–20 mo old mice
single	control βIII-/- *p*	2.42 (1.78, 3.76) 6.42 (1.46, 17.35) 0.077	1.24 (0.11, 7.29) 3.64 (2.60, 7.29) 0.121	2.62 (2.30, 8.82) 4.02 (0.65, 4.96) 0.737
diagonal	control βIII-/- *p*	67.86 (58.86, 75.29) 62.81 (55.97, 67.90) 0.201	72.70 (66.21, 75.23) 55.93 (52.69, 58.31) ***3.77E-07	72.85 (70.06, 76.67) 40.34 (40.01, 41.90) ***1.51E-06
girdle	control βIII-/- *p*	1.82 (1.12, 2.18) 3.46 (1.99, 4.31) **0.005	1.62 (1.11, 3.04) 6.47 (3.85, 10.22) **0.001	1.15 (0.53, 2.93) 3.99 (3.14, 4.32) ***0.00033
lateral	control βIII-/- *p*	1.30 (0.64, 2.11) 2.76 (1.17, 3.26) *0.041	1.63 (0.59, 2.06) 5.42 (2.79, 6.15) ***1.69E-05	2.57 (0.94, 4.31) 8.96 (5.92, 9.49) ***0.00033
three	control βIII-/- *p*	22.88 (17.20, 24.51) 21.29 (15.88, 23.86) 0.484	15.36 (13.04, 24.70) 24.33 (22.61, 29.48) **0.010	14.97 (9.23, 18.63) 38.41 (35.34, 43.08) ***5.23E-05
four	control βIII-/- *p*	2.60 (0.99, 3.52) 1.03 (0, 2.25) 0.192	1.32 (0.42, 4.90) 2.02 (0.37, 3.56) 0.897	1.00 (0.27, 4.68) 3.81 (3.41, 4.31) 0.220

CatWalk analyses was therefore able to identify significant differences in several parameters across the disease course of β-III^−/−^ mice compared to controls providing an unbiased method to reliably monitor progressive ataxia. This ability is key to the search for promising therapeutic approaches as clinically, individuals will only commence treatment at later stages of disease when they are already presenting with significant motor deficits.

### Trimethadione improves interlimb coordination of β-III^−/−^ mice

Based on the evidence supporting aberrant glutamate signalling and disrupted calcium homeostasis as an early feature of β-III spectrin loss (Figs. 1 and 2^2,8,12^;), the CatWalk XT system was used to assess the impact three approved drugs acting on these pathways (riluzole, verapamil and trimethadione) had on the locomotion of overtly ataxic β-III^−/−^ mice. These drugs were chosen based on their reported properties to target the upstream physiological responses associated with β-III spectrin loss, notably impaired glutamate clearance and excessive calcium signalling.

Riluzole, the first of two drugs to be licensed for the treatment of amyotrophic lateral sclerosis (ALS), has been reported to be beneficial in two clinical trials of hereditary ataxia^33^. The mechanism of action is not yet clear but riluzole is reported to inhibit glutamate release^34^ as well as stimulate glutamate clearance^35^. Given the substantive evidence that loss/mislocalization of EAATs, hence impaired glutamate clearance, is a key feature of pathogenesis in SCA5 mouse models riluzole (15 mg/kg/day) was administered to 8-month-old β-III^−/−^ mice in their drinking water for 4 days^2,8,10–12^. However, no significant motor improvement was detected (Table 5).

**Table 5:** Median, quartiles (25th and 75th percentiles) and statistical results of CatWalk XT analyses for 8-month-old βIII^−/−^ mice untreated and administrated riluzole (15 mg/kg/day).

Parameter	Untreated	Riluzole	*p*
Speed (mm/s)	185.33 (160.79, 220.77)	168.98 (155.01, 192.44)	0. 710
Regularity (%)	78.55 (72, 84.86)	76.19 (72.05, 88.27)	1.000
Print position R (mm)	7.85 (5.03, 12.39)	8.18 (4.64, 12.75)	0.902
Print position L (mm)	9.98 (8.72. 10.78)	11.30 (6.16, 12.44)	0. 456
Support Diagonal (%)	57.03 (54.97, 61.41)	54.43 (51.09, 62.78)	0.383
Phase Dispersion RF > LH (%)	7.95 (6.14, 14.71)	12.16 (5.35, 16.25)	0. 805
Phase Dispersion LF > RH (%)	14.02 (12.79, 49.10)	17.66 (6.28, 63.82)	0. 620

RF: right forelimb; LH: left hind limb; LF: left forelimb; RH: right hind limb.

**Fig. 6:**
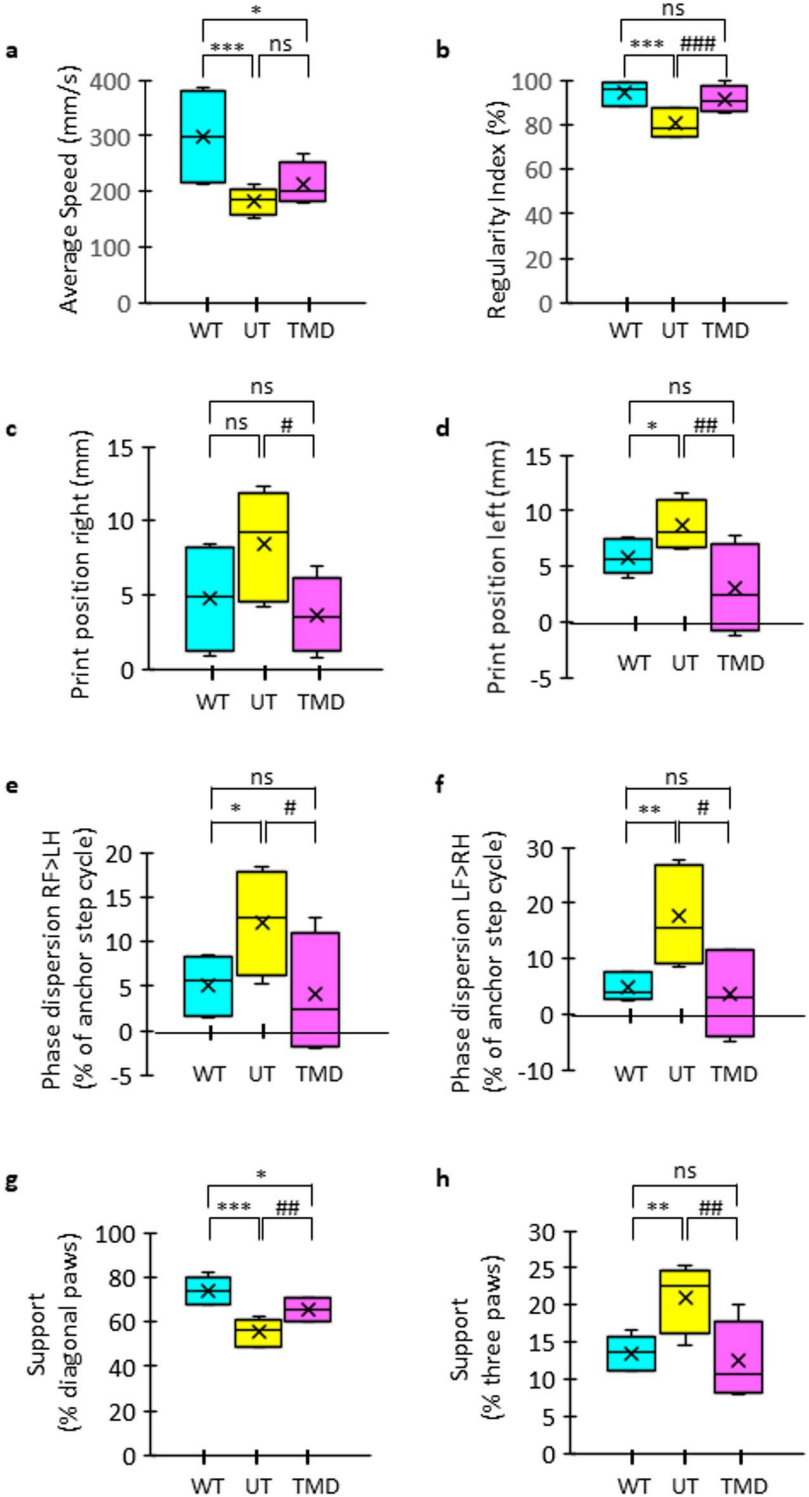
Trimethadione improves interlimb coordination in 8-month- old β-III^−/−^ mice. Quantification of various CatWalk XT parameters for 8-month-old β-III^−/−^ mice administered trimethadione (TMD) in their drinking water. Data from wildtype animals (WT) matched for sex and age to β-III^−/−^ mice plotted alongside for comparison. (**a**) Average body speed. (**b**) Regularity index. (**c**,** d**) Print positions. (**e**,** f**) Phase dispersion. (**g**,** h**) Percentage of total run duration spent supported on diagonal (**g**) and three (**h**) paw combinations. TMD administration significantly improved all parameters, apart from average body speed, for β-III^−/−^ mice when compared to untreated (UT). Greater interlimb coordination with TMD administration also shown by several parameters no longer being significantly different to WT with improvements to average body speed and diagonal support also evident (*N* = 6). Data plotted as median, quartiles (25th and 75th percentiles), minimum and maximum with mean indicated by cross.

Since mibefradil is no longer a clinically used calcium channel blocker two alternative approved voltage-gated calcium channel blockers (verapamil and trimethadione) were selected to be administered to 8-month-old β-III^−/−^ mice. Verapamil, used in the treatment of heart disease, was initially chosen for in vivo application as it is the most similar clinically approved drug to mibefradil since it blocks both L-type and T-type calcium channels. However, it is also reported to block several potassium channels^36^. Therefore, trimethadione (TMD), a selective T-type calcium channel blocker approved for the treatment of absence seizures, was also applied since it does not interact with other channel types. β-III^−/−^ animals administered verapamil (50 mg/kg/day) were lethargic preventing any CatWalk XT analyses being carried out, whereas administration of TMD (200 mg/kg/day), a concentration reported to inhibit absence seizures^37^ resulted in the significant improvement of several CatWalk parameters (RI, print position, phase dispersion and diagonal support) compared to untreated β-III^−/−^, and not significantly different to sex- and age-matched control values except for average body speed and diagonal support (Fig. 6). Together the positive outcomes for both mibefradil in vitro and TMD in vivo indicate modulation of T-type calcium channels has therapeutic potential across the disease course.

## Discussion

The results from this study provide the first molecular evidence of disrupted calcium signalling as an early consequence of β-III spectrin loss. It is also the first study to demonstrate the sensitivity of automated gait analysis being utilised to readily observe changes in motor coordination, even at late stages of disease, in a rodent model of spinocerebellar ataxia. This contrasts with more stressful motor tasks, such as rotarod and elevated beam, which become very challenging and prohibitive for older, more impaired mice. These traditional motor tasks are therefore severely limited in their ability to reliably quantify motor function at later stages of disease, a feature that is central in the search for viable treatment options, especially those that may halt or slow progression. Finally, this study provides the first evidence that T-type calcium channel modulation holds promise as a novel therapeutic option for SCA5 individuals at all stages of the disease. This is because our findings show that mibefradil, which can block T-type calcium channels, rescues the aberrant development of β-III^−/−^ Purkinje cell morphology but also TMD, a selective T-type calcium channel blocker, improves gait kinematics in symptomatic β-III^−/−^ mice. It therefore has wider therapeutic implications for other motor disorders that share aberrant glutamate signalling and/or dysregulated calcium homeostasis as pathogenic pathways.

The CatWalk XT system is routinely the technique of choice to assess the movement of rats before and after spinal cord damage^38,39^. Subsequently it has been used to examine the extent of motor impairment in various rodent models of stroke^40,41^, pain^42–45^, as well as models for various neurodegenerative disorders like ALS, Huntington’s, and Parkinson’s disease^46–49^. To the best of our knowledge, the only other study using the CatWalk XT system to assess spinocerebellar ataxia, utilised a rat model of SCA17^50^. However, for that study, analysis was carried out at a single time-point and therefore the sensitivity of the CatWalk XT system for monitoring the progression of ataxia was not addressed.

The results from the current study clearly demonstrate that the CatWalk XT system is well equipped to detect changes in multiple parameters relevant to a progressive ataxic phenotype with significant changes observed for speed, BOS, print position, regularity index, phase dispersion and support. Here, an increase in both girdle and lateral paw support and phase dispersion of LF > LH were the first parameters to be affected, detected in young mice (6–8-weeks of age) prior to any change in speed across the walkway. The defect in interlimb coordination detected through changes in phase dispersion and support with age appear to arise from a slower transition of opposite hindlimbs resulting in a delay in the placement of hindlimbs relative to the diagonal forepaw, but a significantly earlier placement of hindpaw(s) through the step cycle of hindlimb girdle and ipsilateral front paws. The consequence being a decrease in diagonal and an increase in either lateral, girdle and/or three paw support. It is likely the increased forelimb BOS detected with the CatWalk XT system for 18–20-month-old β-III^−/−^ mice accounts for the previously observed forelimb slips off the elevated beam by 1-year old β-III^−/−^ mice^8^. Together these findings expand upon our initial characterization of the SCA5 mouse model and provide an understanding of the mechanics that underpin the motor deficits observed on traditional motor tasks such as rotarod and elevated beam. Moreover, the CatWalk XT system has the distinct advantage of being less stressful to the animal than these traditional motor tasks, measuring more natural behaviour and providing the ability to quantify multiple features of locomotion, both static and dynamic gait parameters, simultaneously and in an unbiased manner.

In this study riluzole, widely reported to possess anti-glutamatergic properties^34,35,51,52^ was tested based on the substantive evidence implicating glutamate-mediated excitotoxicity in the pathogenesis of SCA5 mouse models^2,8,10–13^. However, administration of 15 mg/kg/day riluzole to the drinking water of β-III^−/−^mice, a concentration within the published range (8–22 mg/kg/day^53,54^), resulted in no motor improvement and there was potentially a trend towards greater impairment following riluzole administration. This could be a consequence of other reported actions of riluzole such as effects on several ion channels, notably inhibition of voltage-gated sodium channels^55^. This would have the potential of further reducing the already diminished β-III^−/−^ Purkinje cell activity^8^ and exacerbating cerebellar dysfunction. Consistent with these observations, a decrease in motor performance was observed for a mouse model of SCA3 treated for 10 months with riluzole^56^. In addition, despite two promising clinical trials for hereditary ataxia, a recent study looking specifically at SCA2 found no improvement with riluzole^57^. This is similar to the MND randomised placebo-controlled clinical trials for riluzole where, although riluzole treatment was associated with an average 10% increase in survival, there was no overall effect on function^58,59^. A retrospective analysis of the clinical trial data has also reported that, irrespective of when treatment begins, riluzole appears to prolong survival in the last clinical stage (stage 4) of ALS, corresponding to nutritional and or substantial respiratory failure, rather than stages 2 and 3^60^. In addition, no improvement of motor function was found in a recent report looking at the effect of riluzole treatment in rodent models of ALS^54^. The results of this present study are therefore in line with these recent MND preclinical studies and SCA2 clinical trial where riluzole fails to improve motor function in β-III^−/−^ mice. Similarly, the fact β-III^−/−^ mice when administered verapamil even for a short period-of-time become lethargic suggests the broad action of this drug may preclude it from being a viable, chronic therapeutic option for cerebellar ataxias. It is feasible that a lower concentration may be better tolerated, but this study already used a concentration half of what is commonly reported^61–63^.

In contrast, trimethadione, a selective T-type calcium channel blocker, was found to improve the interlimb coordination of β-III^−/−^ mice supporting the theory that dysregulated calcium homeostasis, mediated by aberrant activation of low-voltage gated calcium channels, is a key consequence of β-III spectrin loss-of-function. The early increase in CaMKII and GluR1 phosphorylation reported here for young β-III^−/−^ mice may also underpin the previous finding of larger PF-EPSCs in young β-III^−/−^ mice^8^, since enhanced AMPA receptor current conductance occurs with GluR1 phosphorylation^64^. The increased dendritic length and number of branches found in cultured β-III^−/−^ compared to control Purkinje cells could also be due to increased CaMKII activity as overexpression of CaMKIIβ is known to stimulate neurite outgrowth by regulating signalling pathways that influence the cytoskeleton and the transcription of genes involved in neurite outgrowth^29,65^. Moreover, because CaMKIIβ associates with polymerised actin and is enriched where neurite extension is initiated^29^, the disordered dendritic arborization and loss of planarity observed in vivo for β-III^−/−^ Purkinje cells^13^ could arise from changes to this cellular distribution and altered cytoskeletal architecture. The result being neurite extension initiated in an un-regulated manner. The fact mibefradil, a broad calcium channel blocker, can ameliorate aberrant dendritic morphology in vitro provides supporting evidence that dysregulated calcium homeostasis is a factor in early morphological changes. It could be that the resulting thinner dendrites, lead to further abnormal calcium signalling through altered membrane properties making neurites more excitable, impacting activation of low-voltage gated T-type calcium channels.

Motor deficits have previously been alleviated in rodent models of Parkinson’s disease and essential tremor by T-type calcium channels blockers^66–68^ supporting the possibility that these channels present a promising therapeutic target for SCA5. Furthermore, SCA42^69^, epilepsy^70^, autism^71^, essential tremor^72^, and schizophrenia^73^ have all been shown to be associated with mutations in T-type calcium channels. Developing agents that target these channels may therefore hold great potential for various motor disorders with overlapping dysfunctional circuitry. However, this will ultimately depend on whether long-term adverse effects often associated with this class of drug can be minimised^74^.

In conclusion, our results demonstrate that the CatWalk XT system can assess and monitor the age-dependence of motor dysfunction in SCA mouse models. The results provide a strong foundation to further investigate the consequences of disrupted calcium signalling in SCA5 pathogenesis and the potential for T-type calcium channel modulation as a novel therapeutic strategy.

## Electronic supplementary material

Below is the link to the electronic supplementary material.

Supplementary Material 1

Supplementary Material 2

## Data Availability

The data supporting the findings of this study are available from the corresponding author upon reasonable request.
