# Supplementary figures and images for: Automated gait analysis indicates efficacy of T-type calcium channel inhibition for mitigation of disrupted calcium signalling in an SCA5 mouse model
Source: Sci Rep. 2025 Jul 1;15:20990. doi: 10.1038/s41598-025-05511-1 (PMC12216376; doi:10.1038/s41598-025-05511-1)

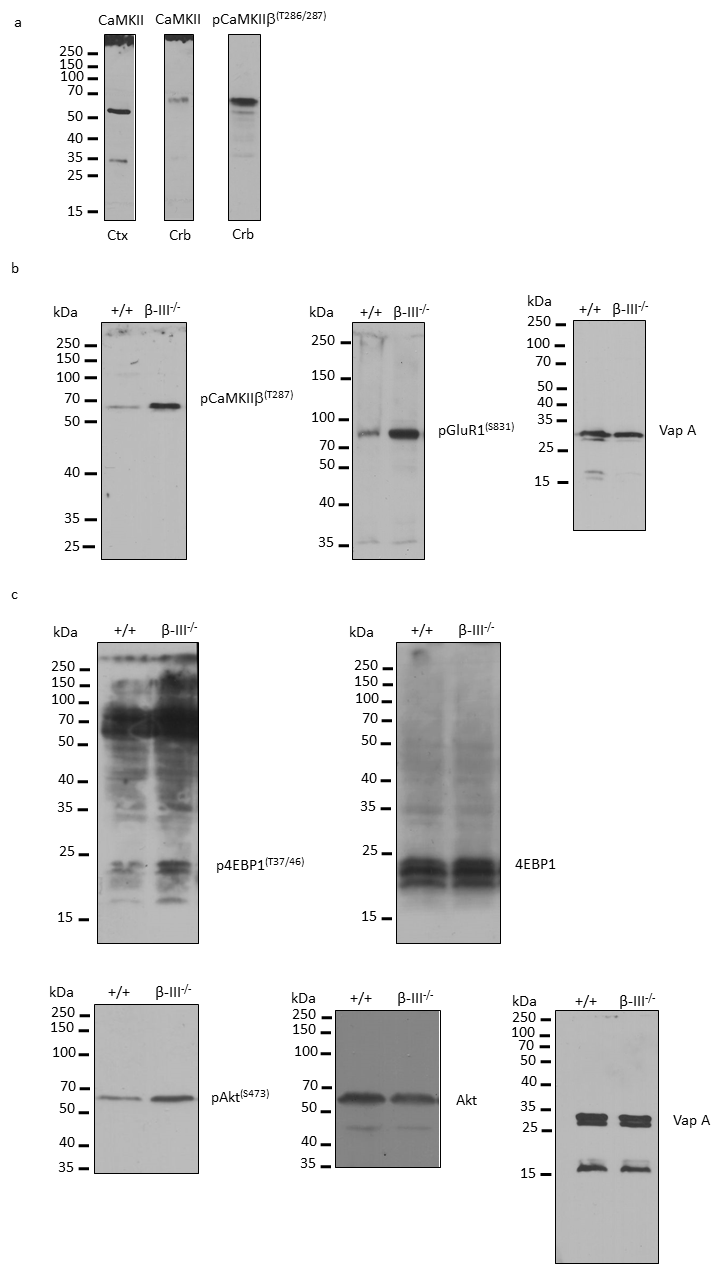

Supplement: Supplementary file 1 — Supplementary Material 1 [file 41598_2025_5511_MOESM1_ESM.tif]
