# Supplementary material for: Automated gait analysis indicates efficacy of T-type calcium channel inhibition for mitigation of disrupted calcium signalling in an SCA5 mouse model
Source: Sci Rep. 2025 Jul 1;15:20990. doi: 10.1038/s41598-025-05511-1 (PMC12216376; doi:10.1038/s41598-025-05511-1)
Supplement: Supplementary file 2 — Supplementary Material 2 [file 41598_2025_5511_MOESM2_ESM.docx]

**Supplementary Figure 1**: Full-length blots for a) cortical and cerebellar lysates from 6-week-old mice, probed with either a pan antibody against CaMKII or phospho-specific CaMKII antibody; b) control (+/+) and β-III^-/-^ cerebellar lysates from 6-week-old mice probed with phospho-specific antibodies against CaMKII and a CaMKII target, GluR1. VapA used as internal loading control; c) control (+/+) and β-III^-/-^ cerebellar lysates from 8-week-old mice examining phosphorylation status of two effectors of a CaMKII activated signalling cascade, Akt (protein kinase B) and 4E-BP1. VapA used as internal loading control.
